# Screening and Identification of DNA Nanostructure Aptamer Using the SELEX Method for ‎Detection of Epsilon Toxin

**DOI:** 10.5812/ijpr-140505

**Published:** 2023-12-08

**Authors:** Nafiseh Shafiei, Hamideh Mahmoodzadeh Hosseini, Jafar Amani, Seyed Ali Mirhosseini, Hanieh Jafary

**Affiliations:** 1 Department of Biology, Science and Research Branch, Islamic Azad University, Tehran, Iran; 2 Applied Microbiology Research Center, Systems Biology and Poisonings Institute, Baqiyatallah University of Medical Sciences, Tehran, Iran

**Keywords:** *Clostridium perfringens*, DNA Aptamer, Epsilon Toxin, ELASA, SELEX, SPR

## Abstract

**Background:**

Epsilon toxin (ETX), produced by *Clostridium perfringens*, is one of the most potent toxins known, with a lethal potency approaching that of botulinum neurotoxins. Epsilon toxin is responsible for enteritis. Therefore, the development of rapid and simple methods to detect ETX is imperative. Aptamers are single-stranded oligonucleotides that can bind tightly to specific target molecules with an affinity comparable to that of monoclonal antibodies (mAbs). DNA aptamers can serve as tools for the molecular identification of organisms, such as pathogen subspecies.

**Objectives:**

This study aimed to isolate high-affinity single-stranded DNA (ssDNA) aptamers against ETX.

**Methods:**

This study identified aptamers using the Systematic Evolution of Ligands by Exponential Enrichment (SELEX) method, enzyme-linked apta-sorbent assay (ELASA), and surface plasmon resonance (SPR) to determine the affinity and specificity of the newly obtained aptamers targeting ETX.

**Results:**

Several aptamers obtained through the SELEX process were studied. Among them, 2 aptamers, ETX clone 3 (ETX3; dissociation constant (Kd = 8.4 ± 2.4E-9M) and ETX11 (Kd = 6.3 ± 1.3E-9M) had favorable specificity for ETX. The limits of detection were 0.21 and 0.08 μg/mL for ETX3 and ETX11, respectively.‎

**Conclusions:**

The discovered aptamers can be used in various aptamer-based rapid diagnostic tests for the detection of ETX.

## 1. Background

*Clostridium perfringens* is a gram-positive, sporulating, rod-shaped bacterium that is heat-resistant and anaerobic. *Clostridium perfringens* resides in water, soil, and the gastrointestinal tracts of various mammals.

*Clostridium perfringens* can produce up to 17 different toxins. Its isotypes include 5 toxinotypes: A, B, C, D, and E, which produce 4 main lethal toxins: Alpha, beta, epsilon, and iota. Strains B and D synthesize the epsilon toxin (ETX) (1).

Epsilon toxin (32.9 kDa) is secreted in the form of an inactive protoxin and can be activated by extracellular serine proteases, such as trypsin, α-chymotrypsin, and k-protease, which eliminate 10-13 N-terminal and/or 22-29 C-terminal amino acid residues. This activated toxin is resistant to proteases in the gastrointestinal tract of mammals (2). Moreover, ETX is one of the most potent toxins known and is responsible for severe diseases in humans and livestock, including enterotoxemia, gastrointestinal, gas gangrene, necrotic enteritis, and enteritis, all of which have significant economic implications worldwide. In addition, ETX is classified as a potential biological weapon, a category B biological agent, and a potential bioterrorism agent. Therefore, rapid detection of ETX is essential (3).

Various techniques have been used to detect ETX, including mass spectrometry (MS) (4, 5), enzyme-linked immunosorbent assay (ELISA) (2, 6), toxin-susceptible cell cultures (7, 8), polymerase chain reaction (PCR) (9, 10), and enzyme immunoassay (EIA) (11, 12). Mass spectrometry is a novel method that overcomes the cross-reactivity issues seen in antibody-based assays. Enzyme-linked immunosorbent assay is a sensitive, quantitative method (6), but it faces challenges related to antibody cross-reactivity and complex matrix effects (4). Cell culture assays using the Madin-Darby canine kidney (MDCK) cell line have been used to detect ETX, but other cell lines are toxin-resistant. Toxin genes are usually amplified using real-time PCR techniques. This is because *Clostridium* toxin genes are mainly located on extrachromosomal elements, and they can be transmitted within different *Clostridium* strains or even other bacterial species. This inter-species genetic transmission is problematic, especially when only genes from a single species are targeted. Enzyme immunoassay methods are commonly used to target toxins because they are sensitive and rapid. However, the number of false-positive results is high (6). Therefore, a quick and simple method is required to detect enteropathogenic bacteria such as C. perfringens. Aptamer-based techniques have been introduced as potential alternatives to traditional methods such as ELISA. Aptamers are single-stranded RNA or DNA oligonucleotide or peptide molecules that form unique 3-dimensional structures due to intramolecular attractions between nucleotides (13). They exhibit high-affinity binding to specific target molecules, including antibodies, metal ions, polysaccharides, lipids, and positively/negatively charged proteins (14, 15). The affinity of aptamers for their targets is equal to or even higher than that of most monoclonal antibodies (mAbs) (16). The dissociation constants (Kd) of aptamer-target complexes range from picomolar to low-micromolar scales (17). Aptamers are highly stable, small, low cost-effective, alkaline pH, heat tolerant, chemically synthesizable, and batch-to-batch reproducible. They also have no restrictions regarding target size or immune response (18-21). DNA aptamers, in particular, offer a new generation of nucleic acid nanostructure-based biomedical tools and diagnostic platforms for molecular recognition (22, 23).

The Systematic Evolution of Ligands by Exponential Enrichment (SELEX) technique is a method to separate particular aptamers that detect aptamers connected to the target from nucleic acid libraries via the immobilization of cell surface proteins on a solid phase (24). This method offers a short selection period, high efficiency, and fast separation time.

## 2. Objectives

This study aimed to design and select high-affinity single-stranded DNA (ssDNA) aptamers against ETX using the SELEX method.

## 3. Methods

### 3.1. Materials

Recombinant ETX was generously provided by the Microbial Science Research Center of Baqiyatallah University. CNBr-activated Sepharose 4B, horseradish peroxidase (HRP)-conjugated streptavidin and bovine serum albumin (BSA) were purchased from Sigma Company (Germany). Biotinylated and non-biotinylated primers from the random DNA oligonucleotide library were purchased from Metabion Company (Germany). Plasmid DNA purification kits were purchased from DNAbiotech Company (Daejeon, Korea). The Luria broth (LB) culture medium was purchased from CONDA Company (Spain), and ampicillin was purchased from Sigma Company (China). The CloneJET PCR Cloning Kit was purchased from Thermo Fisher Scientific Company (USA). All chemicals, including NaCl and HCL, were purchased from Merck Company (Germany).

### 3.2. Amplification and Preparation of Single-Stranded DNA Aptamer Library

The initial ssDNA library was synthesized and purified by polyacrylamide gel electrophoresis (PAGE; Metabion Company). Subsequently, it was diluted to a concentration of 100 pmol following the manufacturer’s instructions. The ssDNA library used 80-mer consisting of the central region randomized sequence of 40 nucleotides flanked by 2 primer-binding hybridization sites. The sequences of the ssDNA library and the primer pairs used are listed in Table 1. The ssDNA library was further diluted to a concentration of 10 pmol. Thereafter, 1 µL of the ssDNA library was amplified in a 25-µL asymmetric PCR reaction, which included 0.8 µL of the forward primer at a concentration of 100 nmol, 1 µL of the reverse primer at 10 nmol, 10 µL of PCR Master Mix, and 12.2 µL of double-distilled water. The amplification process followed a 3-step thermal method, commencing with an initial denaturation at 95°C for 5 min. This was followed by 21 cycles of 95°C for 30 s, 54°C for 30 s, 72°C for 30 s, and a final extension at 72°C for 5 min. The amplified DNA library was run on 2% agarose gel electrophoresis and stored at 20°C until use.

**Table 1.** Oligonucleotides Used in the Systematic Evolution of Ligands by Exponential Enrichment

Name	Oligonucleotides	Length	TM (°C)
**ssDNA library**	5′CCTAACCGATATCACACTCAC(N40)GTTGGTCGTCATTGGAGT ATC-3′	82	-
**Forward primer**	5′-CCTAACCGATATCACACTCAC -3′	21	59
**Reverse primer**	5’-GAT ACT CCA ATG ACG ACC AAC-3’	21	59
**Biotin forward primer**	5’-biotin- CCTAACCGATATCACACTCAC -3	21	59

Abbreviation: ssDNA, single-stranded DNA.

### 3.3. Epsilon Toxin Protein

The concentration of donated ETX was 312 µg/mL.

### 3.4. Affinity Chromatography Column Preparation

Cyanogen bromide-activated Sepharose 4B beads were prepared following the manufacturer’s protocol for affinity chromatography. We added 5 volumes of double-distilled water to every 30 volumes of concentrated Sepharose resin powder, allowing the mixture to form a gel.

Two affinity chromatography columns were prepared as the positive and negative columns in 1.5-mL microfuge tubes. In the first column, 1 nmol of the ETX protein was immobilized onto 30 mg of CNBr-activated Sepharose, which was the positive column for rounds 1-7 of SELEX. In the second column, the ETX-uncoated Sepharose column ‎was used for the negative SELEX process.

### 3.5. The Systematic Evolution of Ligands by Exponential Enrichment Procedure

The SELEX protocol was used to select aptamer candidates for ETX. Initially, 3 nmol of ssDNA aptamers in 300 μL of binding buffer (100 mM NaHCO3, 0.5 M NaCl, PH 8.2) was dissolved at room temperature and then denatured at 95°C for 5 min using a thermal method. Subsequently, they were cooled in an ice bath for 10 min to eliminate any possible double-stranded DNA (dsDNA). The ssDNA aptamer was then added to the negative column and shaken at room temperature for 30 min. The unbound aptamers were collected by centrifugation at 3000 g for 3 min.

The obtained aptamers were then added to a positive Sepharose column containing the ETX protein and incubated for 30 min on a shaker at room temperature. The column was then washed 5 times with 17 mM phosphate-buffered saline (PBS) in rounds 1 - 3 and 7 times in rounds 4 - 6. In round 7, it was washed with 0.02% PBST (Tween 20 + PBS). The stages of the 7 rounds of SELEX for DNA aptamer isolation are listed in Table 2. 

Next, 400 μL of glycine-HCl (100 mM, pH 2.5) was added for 30 min at room temperature with shaking ‎to isolate the oligonucleotides attached to the column. Subsequently, to purify aptamers, the acquired supernatant was precipitated with 2.5 volumes of absolute ethanol, 0.1 volumes of 3 M sodium acetate (PH = 5.5), and 0.5 volumes of 20 mg/mL glycogen, and then incubated at -80°C for 24 h. The mixture was centrifuged at 14 000 rpm for 20 min at 4°C. To remove salts and excess organic matter, the pellet of aptamer was washed with 70% ethanol and air-dried. The final pellet was dissolved in 30 µL of double-distilled water and stored at - 20°C. After each round of SELEX, asymmetric PCR was performed to amplify the aptamer for use as the input in the next cycle. In total, the steps of the positive column were repeated 7 times (Table 2). 

**Table 2.** Details of 7 Rounds of SELEX for Isolation of Single-Stranded DNA Aptamers Against Epsilon Toxin

Round	Amount of ssDNA	Time of Incubation	Washing	Number of Washing	Time of Elution
**1**	3 nmol or 75 μg	30 min	PBS	5	30 min
**2**	2.25 nmol or 60 μg	30 min	PBS	5	30 min
**3**	2.07 nmol or 55 μg	25 min	PBS	5	25 min
**4**	1.88 nmol or 50 μg	22 min	PBS	7	25 min
**5**	1.88 nmol or 45 μg	20 min	PBS	7	20 min
**6**	1.69 nmol or 40 μg	15 min	PBS	7	15 min
**7**	1.5 nmol or 35 μg	15 min	PBS+0.02% Tween20	7	15 min

Abbreviation: ssDNA, single-stranded DNA; PBS, phosphate-buffered saline.

### 3.6. Analysis of Binding Affinity of These Selected Aptamers

An indirect enzyme-linked apta-sorbent assay (ELASA) was conducted to examine the binding affinities. Four rounds of SELEX were haphazardly selected (R1, R3, R5, and R7), and 3 rows of a 96-well microplate were considered as controls (control 1: ETX protein positive [Ag+]/aptamer negative [Apt-]; control 2: ETX protein negative [Ag-]/aptamer positive [Apt+]; control 3: ETX protein negative [Ag-]/aptamer negative [Apt-]).

Initially, the selected aptamers from rounds 1, 3, 5, and 7 were amplified with biotinylated forward primers and non-biotinylated reverse primers at a ratio of 5:1 using asymmetric PCR. For ELASA, 96-well strips were coated with 2 μg of the ETX protein in 100 μL of coating buffer (0.1 M carbonate-bicarbonate buffer, PH 9.6) and left overnight at 4°C. The next day, after washing 3 times with PBS containing 0.05% Tween 20 (PBS + Tween^®^ ‎detergent or PBST), the microplate was blocked with 100 μL of blocking buffer (3% BSA) and incubated for 1 h at 37°C. Then, the wells were washed 3 times with 200 μL of 0.05% PBST, and a mixture of 100 µL of the biotinylated aptamer in binding buffer (100 nM) was added. Plates were incubated at room temperature on a shaker for 3 h and washed 3 times with 200 μL of 0.05% PBST.

Streptavidin-labeled HRP (1: 1000), diluted in PBS, was added to the wells and incubated for 40 min in a shaker incubator at 37°C. Subsequently, each well was washed 5 times with 200 μL of 0.02% PBST, and the color reaction was created using HRP with 3,3′,5,5′-Tetramethylbenzidine (TMB)as a substrate. The reaction was stopped after 15 min with H2SO4 (0.16 M), and the colored product was measured at OD = 450 nm using a microplate spectrophotometer (Bio-Rad PR 3100 TSC 34).

### 3.7. Aptamer Cloning and Sequencing

Based on the binding assay results, the selected oligonucleotides (aptamers) in the seventh round of SELEX were cloned. The CloneJET PCR Cloning Kit (Thermo Fisher Scientific, USA) was used as stated in the manufacturer’s protocol and was transformed into Escherichia coli DH5-α cells that were made competent by incubating in ice-cold 100 mM CaCl2 and then cultured on LB agar.

After an overnight incubation of LB plates containing 80 μg/mL ampicillin at 37°C, colonies containing the vector were selected. Colonies were chosen for screening from the aptamer pool. Subsequently, the confirmed plasmid DNA was extracted using a high-purity plasmid isolation kit (DNABIOTECH, Korea). Plasmids were further amplified through asymmetric PCR using biotinylated primers. The binding affinity of the obtained aptamers was evaluated using ELASA (as previously described). High-affinity colonies were sequenced using a pJET forward primer and analyzed using CLC Sequence Viewer software (Macrogen Company, Korea). The Mfold web server (http://www.unafold.org) was used to predict the secondary structure and minimum free energy.

### 3.8. Determining Parameter Limit of Detection

To assess the potential of the aptamer, the limit of detection (LoD) of the aptamers against ETX was determined. Different concentrations of the ETX protein, ranging from 0.01 to 2 μg/mL, were prepared, and the LoD of the aptamer was calculated during ELASA, as described previously.

 Limit of detection was calculated by LoD = LoB + 1.645 (SD low concentration sample), where LoB = mean blank + 1.645 (SD blank).

### 3.9. Optimizing Aptamer Concentration

To ensure the accurate and efficient identification of the target protein, it is essential to determine the optimal concentration of aptamers. Therefore, 6 different concentrations (3.2, 6.25, 12.5, 25, 50, and 100 nM) of the aptamer were tested using ELASA. The ELASA procedure was conducted as described in section 3.6.

### 3.10. Dissociation Constant Determination Using Surface Plasmon Resonance Analysis

The equilibrium Kd of the selected aptamers was measured using an Autolab ESPRIT surface plasmon resonance (SPR) instrument. During SPR measurement, an acetate buffer (10 mM) was used as the running buffer. Epsilon toxin (50 µg/mL) was immobilized on the sensor surface through covalent binding. The flow rate was maintained at 30 mL/min for 15 min. Different concentrations of candidate aptamers, ranging from 10 to 1500 nM in PBS, were injected at a flow rate of 25 mL/min for 2 min. The regeneration of the aptamer-coated surface was achieved using 1 mM glycine (PH = 2). The Kd values were calculated as the ratio of koff/kon rate constants (koff: Off-rate constant, kon: On-rate constant) using the 1: 1 langmuir interaction analyte model.

### 3.11. Statistical Analysis

The experiment was conducted twice, and the results are expressed as mean ± SD. All statistical analyses were performed using GraphPad Prism version 9 (GraphPad Software, USA).

## 4. Results

### 4.1. Analysis of the Properties of Epsilon Toxin Immobilized on CNBr-Activated Sepharose 4B ‎Beads

CNBr-activated Sepharose 4B was used in the selection procedure due to its speed, reliability, and reproducibility. The target substance, ETX, was attached to the CNBr-activated Sepharose 4B gel. The successful attachment of ETX to the gel was verified by subtracting the absorbance of the supernatant of the Sepharose gel before and after exposure to ETX. The optical density of the supernatant was measured at 280 nm. The absorbance of the supernatant of the Sepharose gel before and after exposure to ETX was 312 and 4 µg/ml, respectively. This observation indicates the successful completion of the stabilization process, with ETX binding effectively to the gel. The gel capacity is approximately 300 μg/mL.

### 4.2. Selection and Amplification of the Aptamer-Specific Pool

**Figure 1.**
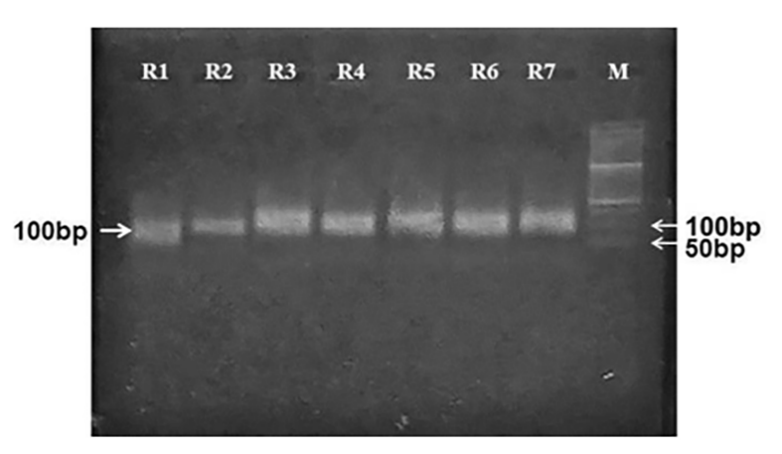
Asymmetric PCR amplification products of each SELEX round. M: 50 bp DNA ladder; 1 - 7: SELEX round numbers

The initial library aptamers were amplified to generate ssDNA aptamers through asymmetric PCR. The properties of aptamer amplification (selection of single-stranded aptamers from the double-stranded population) and the accuracy of their molecular weights on 2% agarose gel electrophoresis were ‎investigated. The results of aptamer amplification of each SELEX round are displayed in Figure 1.‎ 

### 4.3. Binding Affinity of Aptamers in Different SELEX Rounds by the ELASA Method

**Figure 2.**
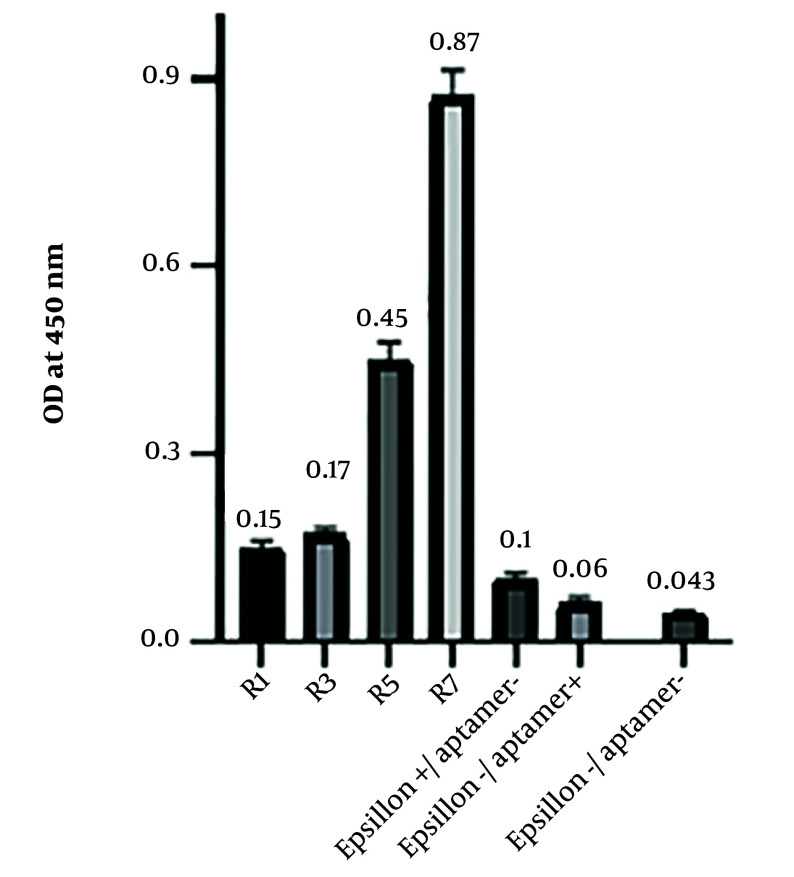
Binding assay of aptamers against the ETX protein in ELASA. The binding affinity of the aptamers for rounds 1, 3, 5, and 7 of SELEX was evaluated and compared with 3 wells, one containing protein, one containing aptamer, and one without aptamer, and protein as negative control groups. Round 7 showed the highest binding affinity

The binding affinity of the aptamers obtained from the first, third, fifth, and seventh rounds was assessed using ELASA. The readout values are shown in Figure 2. The optical density of the seventh round was measured as 0.87 ± 0.008 (Figure 2). Consequently, round 7 was selected for cloning.

### 4.4. Analysis of the Cloning and Sequencing

**Figure 3.**
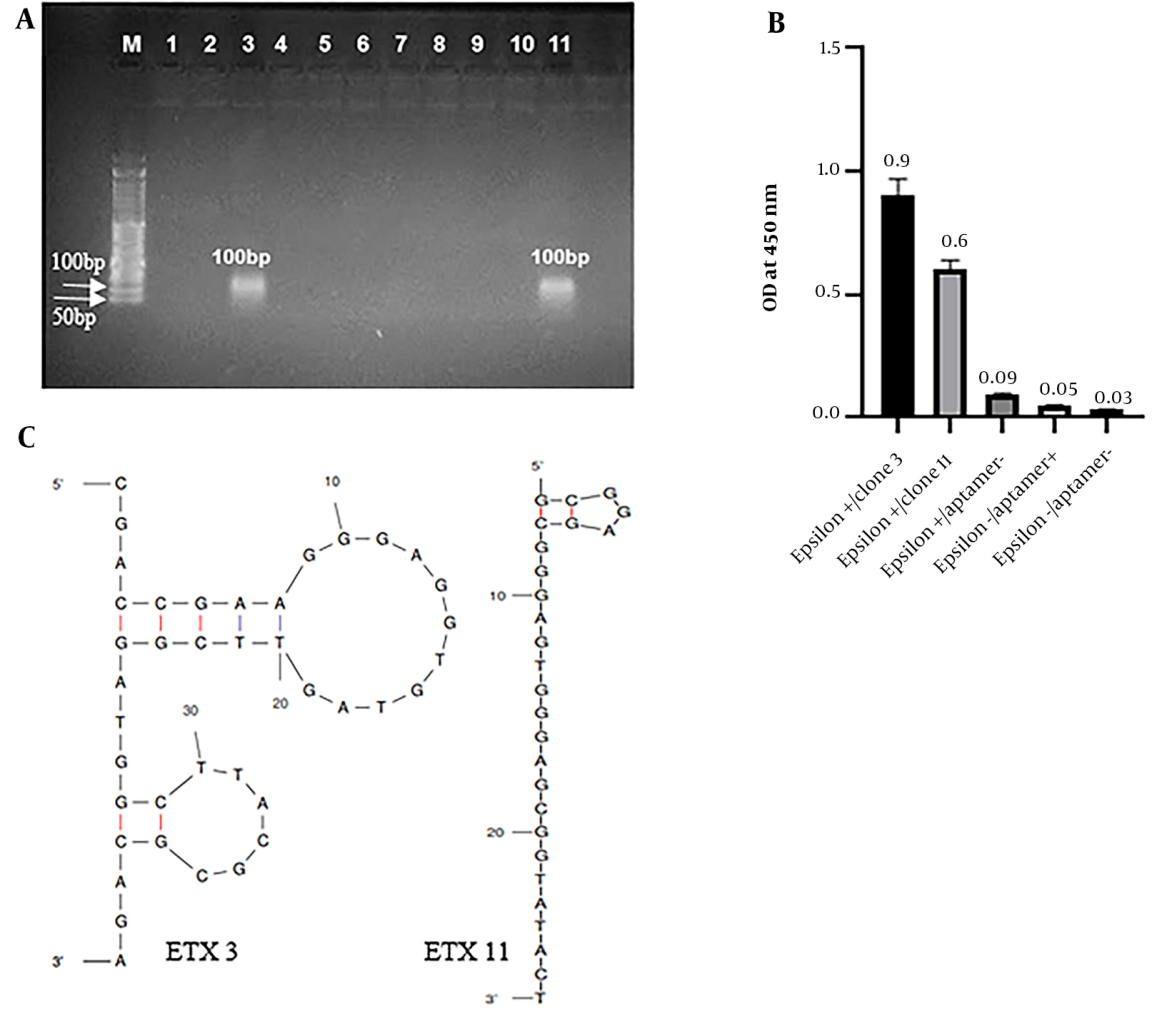
Selection and characterization of the best sequence of aptamers. A, Agarose gel electrophoresis of colony polymerase chain reaction (PCR) products from clones 1 - 11. Clones 3 and 11 were selected and confirmed using colony PCR. M: 50 bp DNA ladder; 1 - 11: Clone 1 to 11; B, enzyme-linked apta-sorbent assay (ELASA) analysis of the 2 cloned aptamer candidates that bind epsilon toxin (ETX). Each of the 2 aptamer sequences is named ETX3 and 11, with optical density (ODs) of 0.9 and 0.6, respectively; C, The secondary structures of aptamer ETX3 and ETX11 were predicted using the UNAFold online program according to the free energy minimization algorithm.

Aptamers obtained from the seventh round of SELEX, which exhibited the highest affinity for ETX, were hosted in the pJET1.2/blunt vector and cloned into E. coli DH5-α. Out of the 11 colonies isolated for sequencing, 2 were confirmed by colony PCR (Figure 3A). 

The binding affinity of the cloned aptamers to ETX was assessed using ELASA. Both aptamers (ETX clone 3 [ETX3] and ETX11) demonstrated almost similar binding affinities. The ELASA results for both aptamers are shown in Figure 3B. 

Finally, sequencing was performed on 2 colonies using pJET forward primers and analyzed using Chromas version 2.13. The sequence of the isolated aptamer (ETX3) is (5’-CGACCGAAGGGAGGTGTAGTTCGGATGGCTTACGCGCAGA-3’), and the sequence of the isolated aptamer (ETX11) is (5’-GCGGAGCGGGAGTGGGAGCGGTATACT-3’). The secondary structure and minimum free energy of the 2 sequences were predicted using the Mfold web server. Figure 3C shows the secondary structures of both obtained aptamers. The ETX3 secondary structure has 2 stem loops, and ETX11 has a linear structure and a pentagonal loop located in the upper part of the ETX11 aptamer sequence. Both aptamers and their 5’ ends were located outside the loop. The minimum free energy was - 4.41 kcal mol^-1^ for aptamer ETX3 and - 0.75 kcal mol^-1^ for aptamer ETX11.

### 4.5. Calculate the Amount of the Limit of Detection

**Figure 4.**
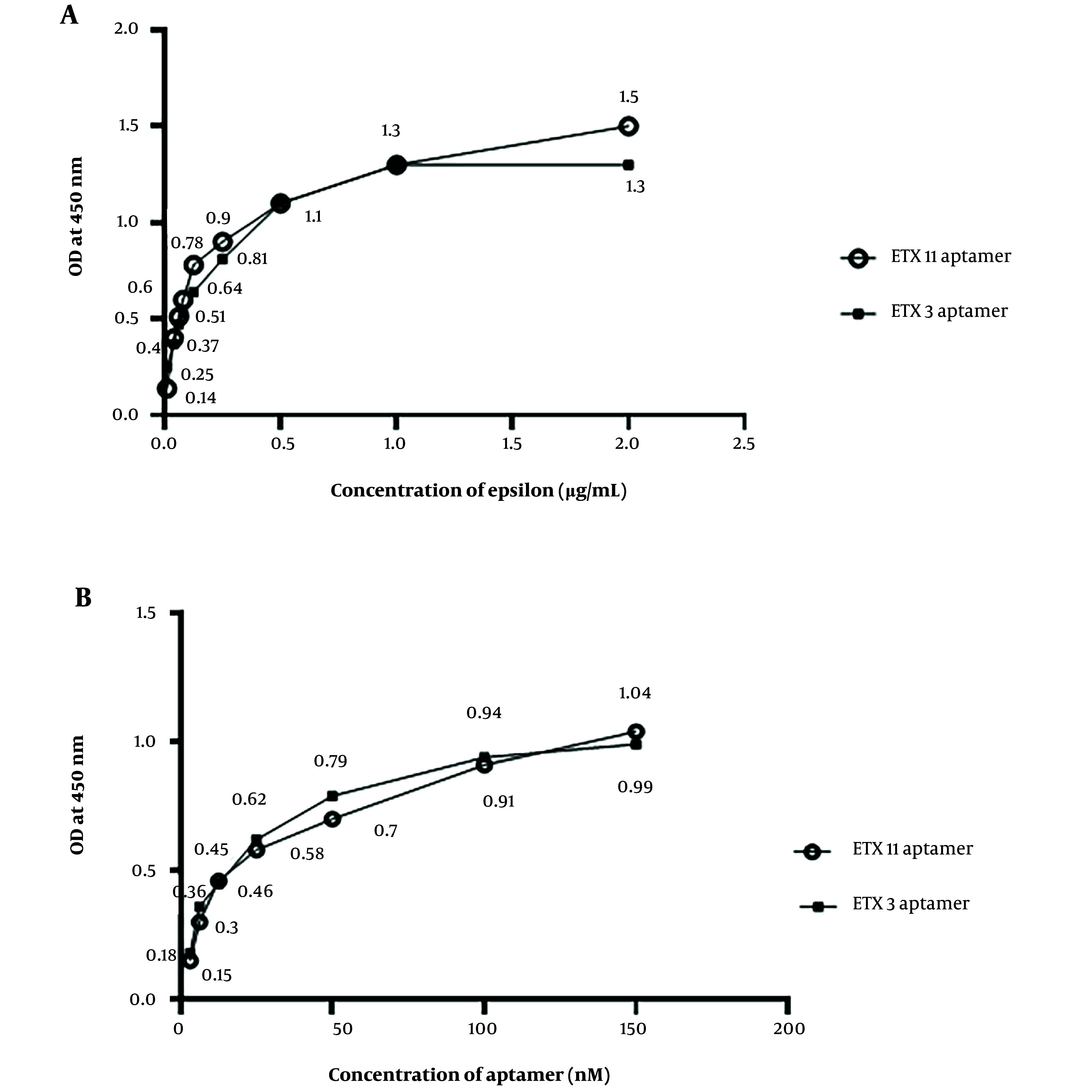
Aptamer sensitivity and optimization using the enzyme-linked apta-sorbent assay method. A, different concentrations of the epsilon protein against the concentrations of epsilon toxin clone 3 (ETX3) and ETX11 aptamers; B, different concentrations of ETX3 and ETX11 aptamers against the concentration of the epsilon protein

Figure 4A shows the aptamer sensitivity assay using different concentrations of the epsilon protein via ELASA. The results revealed that the absorbance value increased with ‎an increasing ETX concentration from 0.01 to 1 µg/mL. There was a tendency for the absorbance value to stabilize at concentrations higher than 1 μg/mL for the ETX3 aptamer and 2 μg/mL for the ETX11 aptamer (Figure 4A). A standard curve was constructed based on these results. The LoD values for ETX3 and ETX11 were estimated to be 0.21 and 0.08 µg/mL, respectively. The data analysis is presented in Table 3. 

**Table 3.** Calculation of the Limit of Detection Based on Epsilon Toxin Clone 3 and Epsilon Toxin Clone 11 Yielded Single-Stranded DNA Aptamers for Epsilon Toxin

Samples	OD			Ave	SD	RSD	RSD%
**ETX3 aptamer**							
2 µg ETX3	1.3	1.25	1.31	1.2866	0.02624	0.020394	2.0394
1 µg ETX3	1.22	1.247	1.291	1.2526	0.02926	0.233594	23.3594
0.5 µg ETX3	1.144	1.18	1.161	0.1616	1.00017	6.189170	618.9170
0.25 µg ETX3	0.81	0.826	0.864	0.8333	0.0226	0.027121	2.7121
0.125 µg ETX3	0.64	0.653	0.627	0.64	0.01061	0.016578	1.6578
0.08 µg ETX3	0.53	0.565	0.598	0.5643	0.027764	0.049200	4.9200
0.06 µg ETX3	0.47	0.472	0.475	0.4723	0.002055	0.004351	0.4351
0.04 µg ETX3	0.374	0.37	0.371	0.371	0.001825	0.004919	0.4919
0.02 µg ETX3	0.31	0.351	0.317	0.326	0.01790	0.05490	5.4907
0.01 µg ETX3	0.25	0.23	0.199	0.2263	0.007525	0.033252	3.3252
Cont.(Ag+/Apt -)	0.046	0.061	0.066	0.0576	0.00849	0.147395	14.7395
Cont.(Ag-/Apt+)	0.074	0.065	0.082	0.0736	0.012028	0.163423	16.3423
Strp+Biotin	0.068	0.09	0.086	0.0813	0.009568	0.117687	11.7687
**ETX11 aptamer**							
2 µg ETX11	1.5	1.526	1.542	1.5226	0.017307	0.01136	1.136
1 µg ETX11	1.30	1.32	1.31	1.31	0.0081649	0.00623	0.0623
0.5 µg ETX11	1.01	1.18	1.06	1.0833	0.0713364	0.06585	6.585
0.25 µg ETX11	0.9	0.926	0.964	0.93	0.0262805	0.02825	2.825
0.125 µg ETX11	0.781	0.765	0.797	0.781	0.01306394	0.01672	1.672
0.08 µg ETX11	0.610	0.603	0.616	0.60	0.0110302	0.01838	1.838
0.06 µg ETX11	0.521	0.506	0.515	0.514	0.006164	0.011992	1.1992
0.04 µg ETX11	0.412	0.410	0.403	0.408	0.0038729	0.009492	0.9492
0.02 µg ETX11	0.302	0.365	0.307	0.324	0.0286006	0.088273	8.8273
0.01 µg ETX11	0.133	0.142	0.168	0.1476	0.0148400	0.100542	10.0542
Cont. (Ag+/Apt -)	0.026	0.072	0.077	0.058333	0.022952	0.393465	39.3465
Cont. (Ag-/Apt+)	0.064	0.066	0.072	0.06733	0.0033993	0.050487	5.0487
Strp+Biotin	0.02	0.05	0.04	0.036	0.0124	0.34444	34.44

Abbreviations: OD, optical density; RSD, relative SD; ETX3, epsilon toxin clone 3; Strp, streptavidin.

Column 1 shows the serial dilution of ETX in the range of 0.01 - 2 µg/µL coated in each row, as indicated in the table, along with controls. The controls contained repeated calculations of (protein+/Apt-), (protein-/Apt+), biotin directly added into the streptavidin-HRP well, and PBST-serum + ETX. Columns 2 - 4 represent the absorbance at 450 nm, column 5 demonstrates the average mean, column 6 demonstrates SDs, and columns 7 and 8 show the relative SD (RSD) and RSD%.

### 4.6. Optimization of Epsilon Toxin Clone 3 and Epsilon Toxin Clone 11 Aptamer Concentrations for Epsilon Toxin Detection

The highest absorption value was observed at 100 nM for both the ETX3 and ETX11 aptamers when tested at different aptamer concentrations against a constant epsilon protein concentration of 2 μg/mL (Figure 4B). 

### 4.7. Dissociation Constant Determination

**Figure 5.**
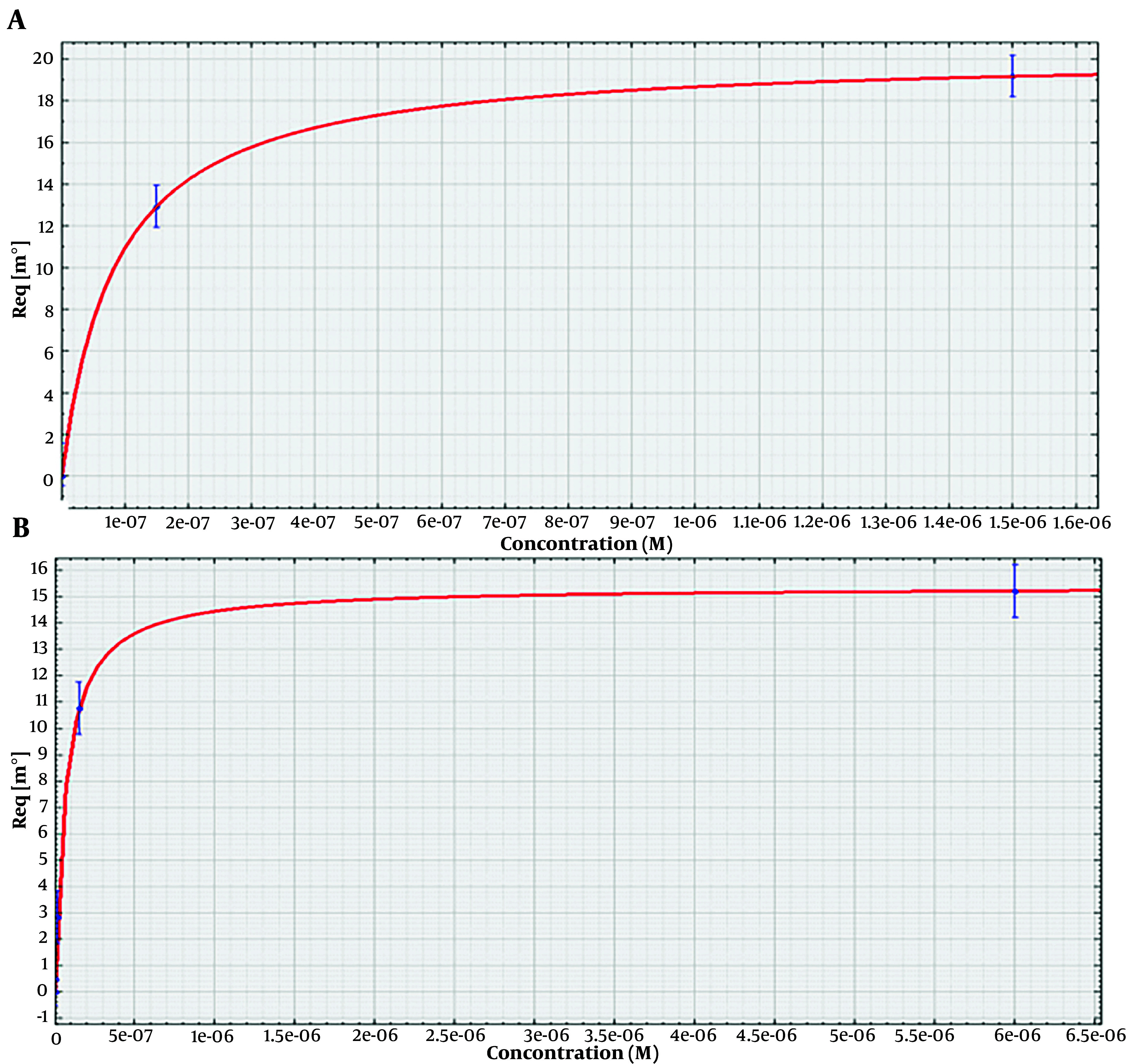
Surface plasmon resonance (SPR) analysis of aptamers for Kd determination. A range of aptamer concentrations (1.5, 15, 150, and 1500 nM) prepared in 10 mM PBST (PH = 7.4) was passed over the surface containing immobilized ETX (50 µg). A, the sensorgram of SPR was used to evaluate the binding affinity between the epsilon protein and the ETX3 aptamer; B, the sensorgram of SPR to evaluate the binding affinity between the epsilon protein and ETX11 aptamer

The surface plasmon resonance analysis of the aptamers is shown in Figure 5. The numerical values of equilibrium Kd were determined using an SPR assay. The dissociation constant was calculated for the binding of ETX3 and ETX11 aptamers to the immobilized epsilon protein. Four different concentrations of each aptamer (ETX3 and ETX11) were used to determine the binding kinetics and calculate the Kd values. The Kd values for ETX3 and ETX11 aptamers were determined to be 8.4 ± 2.4E-9 M and 6.3 ± 1.3E-9 M, respectively.

Since Kd values less than 10 nM indicate high-affinity interactions, the low Kd values obtained indicated strong, high-affinity binding between the aptamers and epsilon protein.

## 5. Discussion

*Clostridium perfringens*, as reported by the Centers for Disease Control and Prevention (CDC), is among the most common causes of foodborne illnesses (food poisoning), leading to nearly 1 million cases in the United States each year. It can infect both humans and animals (25), resulting in symptoms such as diarrhea and stomach cramps within 6 to 24 h after consuming contaminated food. Dehydration is a critical concern in cases of severe diarrhea (26).

Aptamers have been widely used as proper tools in various diagnostic systems, including ELISA, immunocytochemistry, immunofluorescence microscopy, flow cytometry, blotting, and microarray assays (27, 28).

In the present study, a specific ELASA system was developed for the detection of ETX using a specifically developed aptamer. To achieve this, a library of aptamers was generated consisting of oligonucleotides containing a 40-nucleotide random sequence flanked on both ends by 21 nucleotides of the constant sequence.

The length of the random region creates structural diversity in the library (29). Aptamers have been successfully generated from libraries containing random regions ranging from 22 to 220 nucleotides, with an average random region length typically falling in the range of 30 to 80 nucleotides. This allows up to 10^13^ - 10^15^ unique sequences to be screened in a single library (30, 31). The aptamer library’s constant primer regions with a length of 18 to 21 nucleotides act as primers for target-bound sequences during PCR amplification (32).

These constant regions can cause non-specific binding by their nature and increase the incidence ‎of false positives or interfere with binding within the random sequences. Therefore, the sequences should be selected in a manner that does not reduce the overall success of the experiment (33).

In recent decades, further developments and innovations have been made in SELEX. Various novel SELEX technologies have been developed to simplify the process and ameliorate the variety of aptamers (eg, graphene oxide-SELEX, capillary electrophoresis-SELEX, cell-SELEX, and fluorescence-activated cell sorting SELEX). While these novel selection methods were able to obtain several small-molecule aptamers, it is still difficult to screen such compounds because of the challenges of immobilizing molecular particles on solid supports (34). Affinity chromatography was selected as the method for protein immobilization in our study primarily due to its ability to provide acceptable sensitivity and specificity. Additionally, the accuracy and cost-effectiveness of affinity chromatography are comparable to those of other commonly used methods, such as magnetic separation. On the other hand, the reaction components, especially in the columns of chromatography, are highly reproducible (35).

However, one potential limitation of the aptamer selection process is the absence of a counter protein for counter-selection to remove nonspecific binders. This limitation was attributed to the exceptionally unique structural features of ETX (stable tertiary structure, multiple disulfide bonds, specialized cholesterol-binding domain, and amphipathic properties), as well as its potent biological activity (3, 36, 37). Thus, no protein that had a similar epitope structure to ‎epsilon toxin and contaminant properties for cross-target counter selection was found.

Typically, the aptamer pool with the highest affinity for the target molecule is enriched after 5-15 rounds (34). This may lead to some drawbacks, such as the potential loss of scarce sequences, low-affinity aptamers, and PCR bias (38). Based on these results, the specific ssDNA binding to ETX can be effectively enriched through a feasible selection strategy.

Since aptamers with stable structures are more likely to bind to specific targets, a structural stability check is considered a crucial screening step for aptamer candidates (39). Secondary structures typically consist of loops, hairpins, and cruciform structures, resulting in complex 3-dimensional structures that bind to targets through non-covalent bounds (40). Additionally, free energy can be interpreted as the energy released through the bending ‎of a fully unfolded RNA/DNA molecule. A low free energy indicates that a sequence will remain stable within a given structure (39). Consequently, variations in hydrogen bonding, hydrophobic interactions, and salt linkages due to different environmental conditions can influence the secondary structure and cause conformational changes in aptamers, which can affect their affinity for specific targets (41).

Our in silico results predict that aptamer ETX3 may exhibit greater structural stability compared to aptamer ETX11. This prediction is based on the presence of a hairpin structure and a lower minimum free energy of -4.41 kcal/mol. Furthermore, both aptamers (ETX3 and ETX11) demonstrated recognition of ETX with appropriate limits of detection. They exhibited specific binding to ETX with satisfactory Kd values in the micro- to nano-molar range, which is comparable to the binding affinity of many commercially available antibodies.

Our results are consistent with and comparable to those reported in the literature. However, different detection methods were used. For example, Cecile Feraudet-Tarisse et al. studied the affinity of 5 antibodies, PεTX2, PεTX5, PεTX6, PεTX7, and PεTX9, using sandwich EIA (LoD = 0.15 p). Using the SPR biosensor and ETX as antigen, they determined the kinetic parameters of these antibodies to be 9.6 × 10^-9^, 9.8 × 10^- 9^, 1.9 × 10^-9^, 3.7 × 10^-9^, and 2.1 × 10^-9^ M, respectively (42).

In another study, the analytical sensitivity of PCR for ETX genes was determined to be 227 pg/µL (43). A similar approach estimated the LoD of ETX to be approximately 3 ng/mL using ultraperformance liquid chromatography-tandem MS (4). Another detection method is ELISA, in which the LoD for epsilon toxin is reported to be 5.37 ng/mL (44).

When comparing the findings of ELASA and ELISA using commercial kits containing mAbs, it becomes evident that the measured concentrations of ETX in both antibody- and aptamer-based methods are quite comparable. This is because aptamers have a smaller molecular weight than antibodies and can move around contaminated samples more easily to look for their target. Hence, aptamer-based methods can replace antibody-based kits by efficiently identifying biomarkers.

### 5.1. Conclusions

In this study, specific aptamers against ETX were isolated and presented. Their affinity and specificity for the detection of ETX were evaluated using the ELASA method. The Kd values of 8.4 ± 2.4E-9 M and 6.3 ± 1.3E-9 M were calculated for the selected aptamers ETX3 and ETX11, respectively.

This system can be used as a standalone diagnostic method in the laboratory due to its cost-effectiveness, reproducibility, sufficient flexibility, and sensitivity. Moreover, the polyhedral structure of the aptamer suggests an effective approach to enhancing the sensitivity of aptamer-based diagnostic devices, given their high binding affinity to the target protein. Unlike antibodies, the innovative aptamer technique is still in its early stages and requires further improvement to guarantee its widespread use. Using the next generation of high-throughput sequencing and robust nucleic acid chemistry will have a fundamental and sustainable impact on the applicability of aptamer-based methods.

## Data Availability

The dataset presented in the study is available on request from the corresponding author during submission or after publication.
